# Causal relationship between stable angina and bone mineral density: A two-sample bidirectional Mendelian randomization study

**DOI:** 10.1097/MD.0000000000045799

**Published:** 2025-11-07

**Authors:** Yuewei Song, Hao Tian Li, Yaxin Pan, Yanjun Liu, Weidong Sun

**Affiliations:** a Beijing University of Chinese Medicine, Beijing, China; b Wangjing Hospital, China Academy of Chinese Medical Sciences, Beijing, China; c Institute of Basic Theory for Chinese Medicine, China Academy of Chinese Medical Sciences, Beijing, China.

**Keywords:** bone mineral density, causality, Mendelian randomization, single nucleotide polymorphism, stable angina pectoris

## Abstract

This study assesses the causal relationship between bone mineral density (BMD) and stable angina pectoris (SAP) using Mendelian randomization analysis. We obtained genome-wide association study databases for stable angina and BMD, and conducted Mendelian randomization analysis with BMD as the exposure factor and SAP as the outcome. The inverse variance weighted (IVW) method was used as the primary analytical approach, complemented by Cochran *Q* test, the weighted median method, MR-Egger regression, and the simple weighted model to evaluate the robustness and reliability of the results. The IVW results revealed a significant positive correlation between BMD and the incidence of SAP. When BMD was considered as the exposure factor, the analysis indicated an increased risk of SAP with higher BMD. Similarly, when only left heel BMD was considered as the exposure factor, a positive correlation with SAP was observed. However, no significant associations were observed between BMD at other sites and SAP. When SAP is the exposure factor and BMD is the outcome factor, left heel BMD and right heel BMD are both negatively correlated with SAP, while no significant correlation is observed for the remaining sites. There is a positive correlation between BMD and left calcaneal BMD with SAP, whereas SAP exhibits a negative correlation with both left and right calcaneal BMD. These findings are consistent with previous observational studies.

## 1. Introduction

Angina pectoris is commonly defined as chest discomfort induced by physical exertion or emotional distress. When myocardial oxygen supply fails to meet demand, myocardial ischemia occurs, leading to angina pectoris.^[1,2]^ Angina pectoris is categorized into stable and unstable types, with stable angina pectoris (SAP) referring to patients whose chest pain intensity, frequency, nature, and triggering factors remain relatively unchanged over several weeks.^[3]^ In the United States, approximately 10 million adults suffer from SAP, which typically arises when myocardial oxygen supply cannot meet demand, resulting in myocardial ischemia. SAP is associated with an average annual risk of 3 to 4% for myocardial infarction or death.^[4]^ SAP is a typical symptom of stable ischemic heart disease, also known as chronic coronary syndrome, which is a major contributor to disability and dysfunction among the elderly, significantly affecting their mobility and daily lives.^[5]^ Among Americans older than 75, 30% suffer from stable ischemic heart disease, impacting over 3 million elderly individuals,^[6,7]^ and it is a stronger predictor of future disability than acute myocardial infarction.^[8]^ Given this significant disease burden, early prevention, diagnosis of SAP, and exploration of its pathogenesis and related influencing factors are particularly important.

Bone mineral density (BMD) reflects the level of bone mineralization and is directly related to bone stiffness.^[9]^ Currently, various methods are available for assessing BMD, with dual-energy X-ray absorptiometry (DXA) being the most widely used. DXA results are commonly reported using T-scores. T-scores represent the SD of a patient’s BMD compared to the average peak bone mass of healthy young adults of the same sex and ethnicity/race. The World Health Organization has established critical T-score thresholds for defining reduced bone mass and osteoporosis.^[10]^ A T-score between −1.0 and −2.5 is defined as reduced bone mass, and a T-score of −2.5 or below defines osteoporosis.

The association between abnormal bone density and cardiovascular disease has become a research focus in recent years. Significant progress has been made in the diagnosis and treatment of SAP and abnormal bone density. The diagnosis of SAP typically combines noninvasive and invasive tests with patient history.^[11]^ Meanwhile, as a widely used diagnostic tool, bone density has become a research focus in terms of its association with cardiovascular disease risk. Observational studies have found that abnormal bone density may have a causal relationship with cardiovascular diseases such as coronary heart disease, suggesting that bone density and related tests may serve as diagnostic tools for identifying individuals at risk of cardiovascular disease.^[12,13]^ However, the causal relationship between the 2 is not yet clear. Traditional observational designs are inherently limited by confounding factors and reverse causality,^[14]^which obscure the true causal pathways. Cardiovascular disease and osteoporosis share several risk factors, such as age, gender, lifestyle, and chronic inflammatory status,^[15,16]^ which may interfere with the analysis of causality in clinical observational studies. For example, as age increases, the walls of arteries and arterioles thicken and lose elasticity, affecting heart and vascular function.^[17]^ Simultaneously, changes in hormone levels, such as estrogen and testosterone, can also affect bone metabolism, leading to abnormal bone density.^[18]^ In terms of medication, guidelines in the United States and Europe recommend β-blockers and calcium channel blockers (CCBs) as first- or second-line treatments for angina pectoris.^[19,20]^ Studies have shown that certain antihypertensive and vasodilator drugs may affect bone density.^[21,22]^ Research has found that genetic surrogates for angiotensin receptor blockers and thiazide diuretics may have protective effects on bone health, while those for CCBs and potassium-sparing diuretics may have negative effects. Additionally, thiazide diuretics may have a positive effect in reducing fracture risk. In terms of lifestyle, increasing physical activity (PA) can reduce the risk associated with sedentary behavior and cardiovascular disease mortality. This is particularly true for individuals who are sedentary in their daily lives,^[23]^ as increasing moderate-to-vigorous PA can significantly reduce these risks. Exercise is also believed to improve bone density and cardiovascular health.^[24]^ For example, Kitagawa et al found that impact training may be more effective than moderate-to-high-intensity resistance training in improving lumbar spine BMD among postmenopausal women with osteoporosis.^[25]^ Lee et al found that long-term moderate-intensity aerobic exercise also has a positive effect on hindlimb BMD and mitigates age-related trabecular bone loss in the femur.^[26]^ These issues obscure the true causal pathways. Recent advances in Mendelian randomization (MR) have demonstrated its utility in addressing these limitations. For instance, MR analyses have revealed causal effects of sleep traits on angina mediated by cardiovascular risk factors,^[27]^ bidirectional links between Alzheimer disease and prostate cancer,^[28]^ and the impact of aspirin use on physiological outcomes in European populations.^[29]^ These studies highlight MR’s ability to identify cardiovascular risk factors and disentangle complex causal relationships. This study aims to explore the causal relationship between bone density and SAP using MR methods to better exclude confounding factors and obtain a clearer causal relationship,^[30,31]^ providing more accurate evidence for the association between the 2.

## 2. Data and methods

### 2.1. Study design

The basic principles of the MR study design are as follows: instrumental variables are uncorrelated with confounding factors; instrumental variables are associated with the exposure factor; instrumental variables have no direct association with the outcome variable and can only indirectly affect the outcome variable through the exposure factor. This approach is particularly advantageous when examining lifelong exposures and outcomes with delayed clinical manifestations, as it avoids biases from reverse causation and temporal confounding.^[32]^ In this study, the outcome variable is stable angina, the exposure factor is BMD (whole body and various sites), and the instrumental variables are genetic loci associated with BMD, derived from their respective GWAS studies. A two-sample MR design was used to explore the causal association between BMD and stable angina.

**Figure 1.**
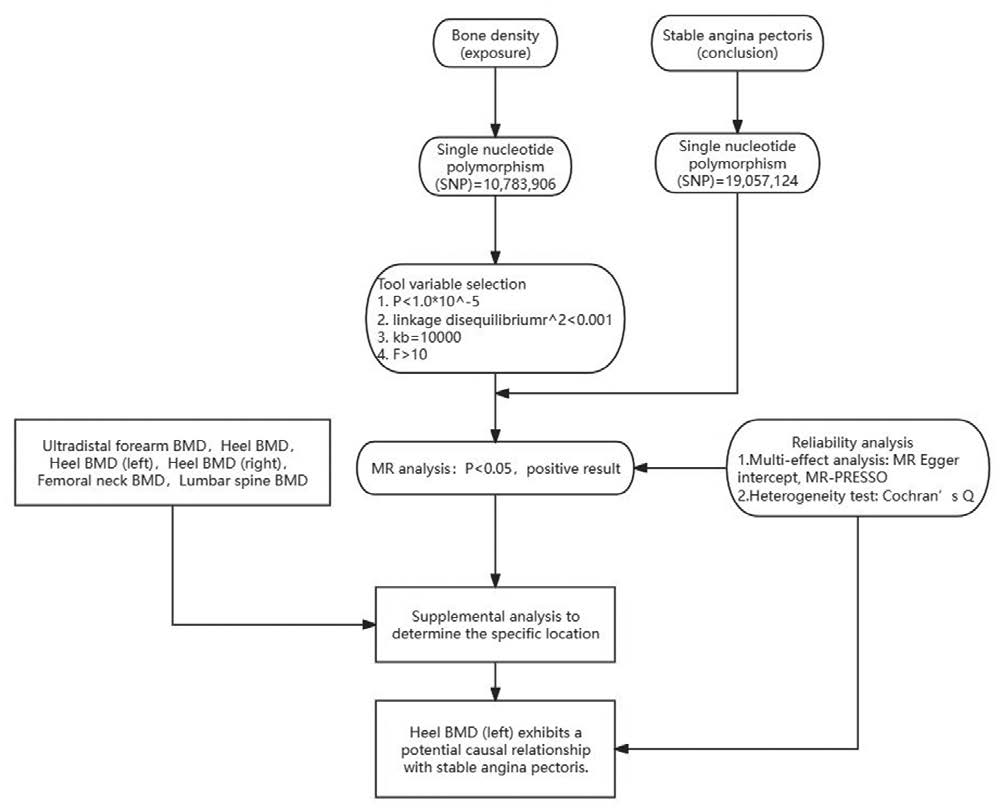
Mendelian randomization (MR) analysis utilizing BMD and heel BMD single nucleotide polymorphisms (SNPs) as instrumental variables to determine the causal impact of BMD on stable angina. BMD = bone mineral density.

The study design is shown in Figure 1. First, we conducted an MR analysis to assess the causal relationship between BMD and stable angina, and performed horizontal pleiotropy tests using “MR Egger” and “MR-PRESSO,” and tested for heterogeneity using “Cochran *Q*.” To validate the reliability of the exposure factor, we used multiple sets of different GWAS data and re-conducted the MR analysis, confirming the causal relationship between heel BMD (left) and stable angina, and performed tests for horizontal pleiotropy and heterogeneity to ensure the reliability of the results. Since the data is de-identified and from publicly available databases, informed consent is not required.

### 2.2. Data sources

The GWAS data used in this study includes publication year, sample size, SNP count, and population information, as detailed in Table 1. We selected databases with the most recent data and the largest sample size. The BMD database published in 2021^[33]^ is from the European Bioinformatics Institute (EBI) data (ebi-a-GCST90014022), which has been integrated into the Integrative Epidemiology Unit (IEU) OPEN GWAS database. This database comprises a sample size of 365,403 individuals of European descent with 10,783,906 SNP markers. For validation purposes, the heel BMD data includes a sample size of 194,398 individuals of European descent with 10,894,596 SNP markers, the heel BMD (left foot) data includes 106,254 individuals of European descent with 10,894,596 SNP markers, and the heel BMD (right foot) data includes 106,251 individuals of European descent with 10,894,596 SNP markers, these markers are from the UK Biobank (UKB) data in the IEU GWAS database (ukb-a-500), (ukb-a-361), and (ukb-a-362). Lumbar spine BMD^[34]^ includes a sample size of 28,498 individuals with 10,582,897 SNP markers, obtained from the IEU database (ieu-a-982). Femoral neck BMD includes a sample size of 32,735 individuals with 9955,366 SNP markers, obtained from the IEU database (ieu-a-982). Distal forearm BMD (ebi-a-GCST90013422) includes a sample size of 21,907 individuals with 11,312,319 SNP markers, from a GWAS study published in Nat Commun in October 2020.^[35]^ The outcome variable, the summary GWAS study results for stable angina (ebi-a-GCST90018915), includes a sample size of 343,026 individuals of European descent with 19,057,124 SNP markers, from a GWAS study published in Nat Genet in October 2021.^[36]^

**Table 1:** Description of GWAS consortia utilized for different phenotypes.

Variable	Sample size	Numbers of SNPs	Population	ID	Consortium	Year
BMD	365,403	10,783,906	European	ebi-a-GCST90014022	NA	2021
Lumbar spine BMD	28,498	10,582,867	Mixed	ieu-a-982	GEFOS	2015
Femoral neck BMD	32,735	10,586,900	Mixed	ieu-a-980	GEFOS	2015
Ultradistal forearm BMD	21,907	11,312,319	European	ebi-a-GCST90013422	NA	2020
Stable angina pectoris	343,026	19,057,124	European	ebi-a-GCST90018915	NA	2021
Heel BMD	194,398	10,894,596	European	ukb-a-500	UKB	2017
Heel BMD (left)	106,254	10,894,596	European	ukb-a-361	UKB	2017
Heel BMD (right)	106,251	10,894,596	European	ukb-a-362	UKB	2017

BMD = bone mineral density, GEFOS = genetic factors of osteoporosis, GWAS = Genome-Wide Association Study, SNP = single nucleotide polymorphism, UKB = UK Biobank.

### 2.3. Instrument variable selection

Firstly, single nucleotide polymorphisms (SNPs) that were significantly associated with BMD (*P* < 5 × 10^−8^) were screened from the database. These SNPs were then used as instrumental variables to evaluate the causal relationship between exposure and outcome in the MR analysis. This was done if their pairwise correlation coefficient (*r*^2^) was <0.001 and their distance exceeded 10,000 kb. PhenoScanner was used to screen and remove confounding factors (no confounding factors were found in this study). The strength of the instruments was estimated using the *F*-statistic, with *F*-statistics <10 considered to indicate weak instrument bias and thus were excluded.^[37,38]^

### 2.4. Main outcome measures and statistical analysis

In the MR analysis, the inverse variance weighted (IVW) method was used as the primary approach to explore the relationship between BMD and BMD of specific sites with stable angina.^[39]^ BMD and BMD of specific sites from different databases were used as exposure factors, and various methods such as MR-Egger, weighted median, simple mode, and weighted mode were applied to test the reliability and stability of the results. All statistical analyses were conducted in the R software environment (version 4.3.1; R Core Team, R Foundation for Statistical Computing, Vienna, Austria) using the “TwoSampleMR” (version 0.5.6) and “MendelianRandomization” (version 0.5.1) packages. Statistical significance was set at *P* < .05.

## 3. Results

### 3.1. Results of MR analysis

**Figure 2.**
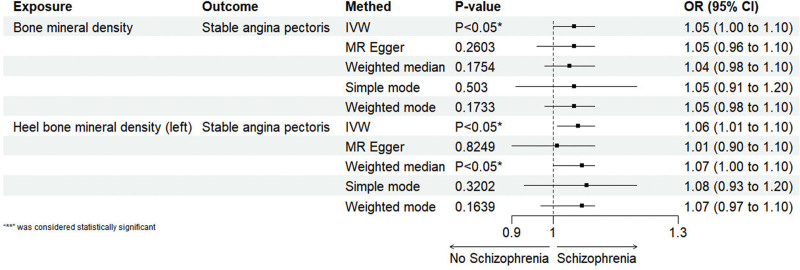
Mendelian randomization (MR) analysis results of BMD and heel BMD (left) single nucleotide polymorphisms (SNPs) in patients with stable angina using 5 methods. BMD = bone mineral density.

The correlation between BMD (various sites) and stable angina was investigated using MR analysis, and the results are shown in Table 2. When bone density was considered as the exposure factor, the analysis indicated an increased risk of stable angina (IVW odds ratio: [OR] = 1.05; 95% confidence interval [CI] = 1.00–1.10; *P* < .05). Similarly, when only heel bone density (left) was used as the exposure factor, we found a positive correlation with the development of stable angina (IVW: OR = 1.06, 95% CI = 1.01–1.12; *P* < .05). An increase of 1 SD in BMD was associated with a 5% increase in stable angina (IVW: OR = 1.05; 95% CI = 1.00–1.10; *P* < .05), and an increase of 1 SD in heel BMD (left) was associated with a 6% increase in stable angina (IVW: OR = 1.06, 95% CI = 1.01–1.12; *P* < .05) as shown in Figure 2.

**Table 2:** Results of Mendelian randomization (MR) analysis of the effects of bone mineral density (BMD) and specific-site BMD on stable angina pectoris.

IVW	ID	SNPs	Beta	Standard error	OR (95% Cl)	*P*-value
BMD	ebi-a-GCST90014022	437	0.0506	0.0233	1.0519 (1.0049–1.10103)	.0299 *P* < .05*
Lumbar spine BMD	ieu-a-982	23	0.0285	0.0337	1.028901 (0.9632–1.0991)	.3974
Femoral neck BMD	ieu-a-980	21	0.0461	0.0555	1.0472 (0.9393–1.1674)	.4060
Ultradistal forearm BMD	ebi-a-GCST90013422	12	−0.0222	0.0363	0.9781 (0.9109–1.0503)	.5419
Heel BMD	ukb-a-500	240	0.0224	0.0252	1.0227 (0.9734–1.0744)	.3732
Heel BMD (left)	ukb-a-361	132	0.0603	0.0254	1.0622 (1.0107–1.1163)	.0173 *P*< .05*
Heel BMD (right)	ukb-a-362	136	0.0331	0.0252	1.0337 (0.9839–1.0859)	.1882

BMD = bone mineral density, GWAS = Genome-Wide Association Study, IVW = inverse variance weighted, MR = Mendelian randomization, SNPs = single nucleotide polymorphisms.

* Statistical significance.

The results showed no significant association between distal forearm BMD, lumbar spine BMD, femoral neck BMD, and heel BMD (right) with stable angina (*P* > .05), which was not statistically significant.

The results of the univariate MR analysis showed no evidence of potential weak instrument bias (all *F*-statistics > 10). The MR-Egger analysis of BMD showed no horizontal pleiotropy (intercept *P* > .05), but the MR-Egger analysis of heel BMD (left) indicated the presence of horizontal pleiotropy (intercept *P* < .05). The use of “MR-PRESSO” detected the presence of outliers, and after removing these outliers, 4 out of 136 SNPs were removed (“rs2306363,” “rs2908007,” “rs4790881,” “rs57332886”), leaving 132 SNPs. The subsequent MR-Egger analysis showed no horizontal pleiotropy (*P* > .05), and the causal relationship remained significant (*P* < .05). A test for heterogeneity between BMD and heel BMD (left) revealed the presence of heterogeneity in both (*P* < .001). Despite some heterogeneity, it did not affect the results of the IVW method, and our conclusions are reliable. Sensitivity analysis was performed using the leave-one-out method (Fig. S1, Supplemental Digital Content, https://links.lww.com/MD/Q609) to assess the potential association between BMD, heel BMD (left), and stable angina. As shown in (Fig. S1A, Supplemental Digital Content, https://links.lww.com/MD/Q609), the exclusion of any single SNP did not result in a significant change in the overall error lines. All error lines for BMD were centered on the right side of 0, and all error lines for heel BMD (left) were on the right side of 0, demonstrating the robustness of the results. The funnel plot (Fig. S1B, Supplemental Digital Content, https://links.lww.com/MD/Q609) showed that all points for BMD were evenly distributed on both sides of the effect line, and after the removal of outliers and reanalysis for heel BMD (left), all points were evenly distributed on both sides of the effect line, indicating no publication or other biases. The scatter plot (Fig. S1C, Supplemental Digital Content, https://links.lww.com/MD/Q609) had the SNP’s effect on BMD and heel BMD (left) on the x-axis and the SNP’s effect on stable angina on the y-axis. The ratio of the 2 effects reflects the effect of exposure on the outcome, i.e., the slope of the line. The results showed that the lines overall were sloping upwards, indicating a positive correlation between BMD, heel BMD (left), and stable angina. Each horizontal solid line in the forest plot (Fig. S1D, Supplemental Digital Content, https://links.lww.com/MD/Q609) represents the results estimated from various SNPs using the Wald ratio method, and the red line segment represents the overall results and CIs. The results showed that both BMD and heel BMD (left) IVW reached significance, indicating a positive correlation. In summary, these methods all confirmed the reliability of our results.

### 3.2. Results of reverse MR analysis

**Figure 3.**
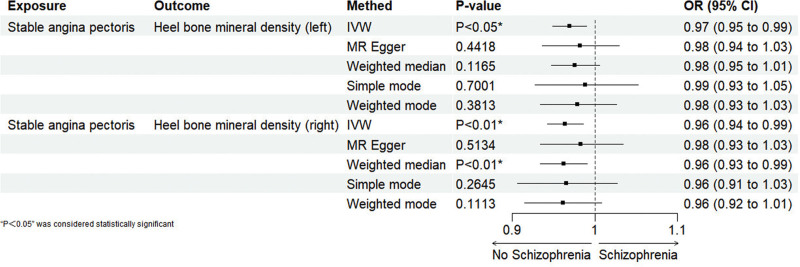
Reverse MR analysis results chart. MR = Mendelian randomization.

The correlation between stable angina and BMD (various sites) was investigated using MR analysis, and the results are shown in Table 3. When stable angina was considered as the exposure factor, the analysis revealed a significant negative correlation with left heel BMD (IVW: OR = 0.97; 95% CI = 0.95–0.99; *P* < .05), and a significant negative correlation with right heel BMD was also identified (IVW: OR = 0.96; 95% CI = 0.94–0.99; *P* < .05) as shown in Figure 3. Indicates that patients with stable angina are more likely to experience a decrease in bone mass in 1 heel bone.

**Table 3:** Results of Mendelian randomization (MR) analysis of the effects of stable angina pectoris on BMD and specific-site BMD.

IVW	ID	SNPs	Beta	Standard error	OR (95% Cl)	*P*-value
BMD	ebi-a-GCST90014022	41	−0.018	0.0094	0.9822 (0.9642–1.0005)	.0569
Lumbar spine BMD	ieu-a-982	45	0.0269	0.03	1.0272 (0.9686–1.0895)	.3704
Femoral neck BMD	ieu-a-980	44	0.0018	0.0217	1.0018 (0.9601–1.0453)	.9348
Ultradistal forearm BMD	ebi-a-GCST90013422	44	0.0214	0.0262	1.0216 (0.9705–1.0754)	.4144
Heel BMD	ukb-a-500	49	−0.0161	0.012	0.9840 (0.9611–1.0074)	.1787
Heel BMD (left)	ukb-a-361	49	−0.0311	0.0108	0.9693 (0.9490–0.9901)	.0040 *P*<.01*
Heel BMD (right)	ukb-a-362	49	−0.0368	0.0118	0.9639 (0.9419–0.9864)	.0018 *P*<.01*

BMD = bone mineral density, IVW = inverse variance weighted, MR = Mendelian randomization, SNPs = single nucleotide polymorphisms. *P < .01.

The results indicate no significant association between stable angina and BMD, distal forearm BMD, lumbar spine BMD, femoral neck BMD, and heel BMD (*P* > .05), which is not statistically significant.

The results of the univariate MR analysis show no evidence of potential weak instrument bias (all *F*-statistics > 10). The MR-Egger analysis of left heel BMD and right heel BMD shows no horizontal pleiotropy (intercept *P* > .05). The heterogeneity test for left heel BMD and right heel BMD reveals heterogeneity in both (*P* < .05). Despite some heterogeneity, it does not affect the results of the IVW method, and our conclusions remain reliable. Sensitivity analysis using the leave-one-out method (Fig. S2, Supplemental Digital Content, https://links.lww.com/MD/Q609) was conducted to assess the potential association between stable angina and left heel BMD and right heel BMD. As shown in (Fig. S2A, Supplemental Digital Content, https://links.lww.com/MD/Q609), the exclusion of any single SNP does not significantly change the overall error lines, and all error lines for left heel BMD and right heel BMD are on the right side of 0, demonstrating the robustness of the results. The funnel plot (Fig. S2B, Supplemental Digital Content, https://links.lww.com/MD/Q609) shows that all points for left heel BMD and right heel BMD are evenly distributed on both sides of the effect line, indicating no publication or other biases. In the scatter plot (Fig. S2C, Supplemental Digital Content, https://links.lww.com/MD/Q609), the horizontal axis represents the effect of SNPs on stable angina, and the vertical axis represents the effect of SNPs on left heel BMD and right heel BMD. The ratio of the 2 effects reflects the effect of exposure on the outcome, i.e., the slope of the line. The results show that the lines for different algorithms are generally upward sloping, indicating a negative correlation between stable angina and left heel BMD and right heel BMD. Each horizontal solid line in the forest plot (Fig. S2D, Supplemental Digital Content, https://links.lww.com/MD/Q609) represents the results estimated from various SNPs using the Wald ratio method, and the red line segment represents the overall result and CI. The results show that the IVW for left heel BMD and right heel BMD both reach significance, indicating a negative correlation. In summary, these methods all confirm the reliability of our results.

## 4. Discussion

BMD, a key indicator for assessing skeletal health, is influenced by various factors, such as chronic kidney disease (CKD) Mineral and Bone Disorder (CKD-MBD) in patients with CKD,^[40]^ and is often accompanied by vascular calcification.^[41]^ Research has also revealed the impact of specific lipid metabolites on BMD.^[42]^ Abnormal BMD can lead to an increased incidence of fractures.^[43]^ SAP, one of the main symptoms of ischemic heart disease, arises from an imbalance between myocardial oxygen supply and metabolic demand. Its pathogenesis is complex, involving factors such as coronary artery stenosis, microcirculatory changes, and coronary artery spasm. Currently, pharmacological therapy and percutaneous coronary intervention (PCI) are the primary treatments for stable angina, aimed at controlling symptoms, improving quality of life, and preventing myocardial infarction to reduce mortality. Guidelines recommend anti-anginal drugs as first-line treatment, with β-blockers, CCBs, or long-acting nitrates being the preferred options for symptom relief, and PCI is reserved for patients who remain symptomatic after first-line treatment.^[44]^ Angina relief is the main reason for percutaneous revascularization (PCI) in patients with stable coronary artery disease.^[45]^ For diagnosis, guidelines suggest a comprehensive approach including symptoms, imaging studies, coronary flow reserve, calcium scoring, coronary CT angiography, and coronary magnetic resonance imaging. Among these, invasive coronary angiography is particularly significant for the diagnosis and treatment of stable angina. In patients with chronic coronary disease who continue to experience worsening symptoms or functional status despite optimal medical therapy (GDMT), invasive coronary angiography is recommended to guide treatment decisions, with the goal of improving angina symptoms. The diagnosis and treatment of stable angina remain active areas of research and clinical challenge. In contrast, the diagnosis of BMD primarily relies on dual-energy X-ray absorptiometry (DXA), which studies have shown to be the most accurate and reliable method for measuring BMD, diagnosing osteoporosis, and monitoring BMD changes, thereby considered the gold standard in the field.^[46]^ Recent research has found that BMD is not simply a protective factor; excessively high BMD may also lead to issues such as vascular calcification, thereby increasing the risk of cardiovascular disease.^[47]^

MR leverages genetic variants as instrumental variables to effectively reduce interference from confounding factors and reverse causality commonly observed in traditional observational studies, thereby enabling more reliable causal inference.^[32]^ This study revealed a positive association between BMD and SAP through MR analysis, indicating that elevated BMD correlates with an increased incidence of stable angina. Elevated BMD is defined as a significant rise in bone mineral content, such as calcium and phosphorus, compared to age- and sex-matched healthy populations. This phenomenon can be identified via imaging techniques like dual-energy X-ray absorptiometry, DXA scanning, and is characterized by T-scores or Z-scores exceeding normal reference ranges. Its clinical definition and implications require comprehensive evaluation within specific diagnostic contexts. In contrast to osteoporosis, elevated BMD is relatively uncommon and exhibits greater etiological and phenotypic heterogeneity. It frequently manifests as a distinctive feature of specific pathological conditions, including but not limited to abdominal aortic calcification, scoliosis, osteoarthritis, renal osteodystrophy with diffuse bone metastases,^[48]^ osteopetrosis, and pseudohypoparathyroidism. Consequently, precise differentiation of its underlying pathogenic mechanisms is imperative,^[49]^ bone density elevation can be divided into pathological and physiological bone density elevation, the former mostly occurs in bone tumors,^[50]^ bone metabolism disorders, and bone injuries, while the latter is more common in adolescent growth and development, exercise,^[51]^ and high calcium intake, all of which are true bone density elevation, whereas pseudo bone density elevation is often seen in imaging artifacts and measurement site errors. Among true bone density elevations, both high calcium intake and hyperparathyroidism are associated with elevated blood calcium levels, and studies have shown that higher calcium levels can lead to calcium deposition causing atherosclerosis,^[52]^ which in turn can induce coronary artery stenosis, affecting myocardial blood supply and oxygenation, resulting in stable angina. High calcium levels can also lead to an increase in intracellular calcium ion concentration, affecting the electrophysiological characteristics of heart cells, leading to an increased risk of arrhythmias,^[53]^ and at the same time affecting the heart’s autonomic nervous system, leading to enhanced sympathetic nerve activity and reduced vagus nerve activity, thus affecting the heart’s rhythm and conduction, which also increases the risk of arrhythmias. Both can lead to myocardial ischemia, triggering stable angina. Additionally, some studies have found that the formation of bone spurs and vascular calcification are related to the occurrence of cardiovascular diseases,^[54]^ particularly in patients with diffuse idiopathic skeletal hyperostosis, suggesting that bone hyperplasia lesions such as bone spurs may be associated with an increased risk of cardiovascular disease. All of the above indicate that bone density is not a simple protective factor; excessively high bone density may also lead to vascular calcification^[38]^ and arrhythmias, thus increasing the risk of cardiovascular disease, which is consistent with our research findings. Therefore, it is crucial for clinicians to distinguish between physiological and pathological causes of BMD elevation. Physiological reasons may include normal growth and development, regular PA, and adequate calcium intake. On the other hand, pathological reasons include conditions like osteopetrosis, Paget disease, and hyperparathyroidism, which can lead to abnormal calcium metabolism and potential cardiovascular complications. For patients with physiological BMD elevation due to normal aging or exercise, it is recommended to maintain a healthy lifestyle and regularly monitor BMD and cardiovascular health. For patients with pathological BMD elevation, the underlying disease should be addressed, and potential cardiovascular complications should be closely monitored.

This association also exists between heel BMD (left) and stable angina. As an important weight-bearing part of the human body, the heel bone’s changes in bone density may more directly reflect the overall health of the skeletal system. Traditional dual-energy X-ray absorptiometry (DXA) often measures bone density at the lumbar spine and hip,^[55]^ while studies have shown that heel BMD (H-BMD) is a useful surrogate indicator for hip BMD.^[56]^ heel quantitative ultrasound has been used to predict the risk of total fractures and hip fractures in both men and women.^[57]^ When it is difficult to measure BMD due to spinal deformities through quantitative computed tomography (CT) or other methods, the calcaneus can be used as an additional site to assess bone density and evaluate patients’ bone loss.^[58]^ Mészáros et al measured the bone density of the left and right calcaneus and femoral neck based on the dominant hand side and found that the heel bone density of the non-dominant side was significantly higher than that of the dominant side in both sexes.^[59]^ Considering the proportion of left and right dominant sides in the general population, this finding supports why the increased left heel bone density is more significantly associated with the increased risk of stable angina, while the right heel did not show a similar positive relationship. The heel is more prone to abnormal bone density under long-term mechanical stimulation, such as Achilles tendinopathy and bone spur formation, these pathological changes lead to an imbalance of calcium salt, increasing the risk of coronary calcification, and subsequently triggering stable angina. This conclusion is consistent with the positive correlation between BMD and stable angina found in this study, further emphasizing the importance of distinguishing the causes of BMD elevation in clinical practice and the necessity of taking corresponding intervention measures for different reasons.

The results of the reverse MR analysis indicate that stable angina is negatively correlated with heel BMD (left) and heel BMD (right), meaning that patients with stable angina are more likely to experience a decrease in heel bone mass or osteoporosis. This finding aligns with the conclusions of previous studies and further enriches our understanding of the complex relationship between osteoporosis and cardiovascular diseases. Previous research generally considers osteoporosis and bone mass loss to be risk factors for cardiovascular diseases,^[60]^ speculating that they may promote the occurrence and development of cardiovascular diseases through common pathophysiological mechanisms such as inflammation, oxidative stress, and vascular calcification. However, there is some controversy and disagreement about the extent to which low bone density may be associated with an increased risk of cardiovascular disease.^[61]^ This study, starting from the perspective of reverse causality, provides new evidence indicating that stable angina, a cardiovascular disease itself, may also affect bone density, leading to a decrease in bone mass. This bidirectional relationship suggests that there may be a more complex interactive mechanism between osteoporosis and cardiovascular diseases. Not only can osteoporosis increase the risk of cardiovascular diseases, but cardiovascular diseases themselves may also affect bone health through certain pathways. The impact of stable angina on bone density can be considered in the following aspects: on one hand, patients’ mobility is reduced, and angina, as an important manifestation of cardiovascular disease, often affects normal PA and exercise, leading to a decrease in bone density in angina patients.^[62]^ On the other hand, the use of medications for stable angina, such as CCBs, has been found to have a negative impact on bone health genetically through target Mendelian analysis.^[20]^

Regarding the influence of confounding factors, MR reduces confounding effects through genetic instrumental variables. However, residual pleiotropy or unmeasured factors may still impact observed associations. For example, cardiovascular medications such as thiazide diuretics and β-blockers may increase BMD in a dose-dependent manner and reduce fracture risk.^[63,64]^ Additionally, systemic conditions like chronic inflammation or metabolic syndrome may concurrently disrupt bone metabolism and vascular health.^[65,66]^ Notably, a study found an inverse correlation between cardiovascular disease prevalence and BMD levels in male patients with type 2 diabetes.^[67]^ Nonetheless, our repeated MR analyses consistently identified associations in both left and right heel BMD, further confirming the robustness of our findings. Future studies could integrate multivariable MR analyses or clinical cohort evidence to clarify causal relationships and underlying mechanisms.

In summary, the results of this study not only support the previous research conclusions about the correlation between osteoporosis and cardiovascular diseases but also further supplement and enrich the complexity of the relationship between the 2. Future research should continue to delve into the mutual mechanisms between osteoporosis and cardiovascular diseases in order to provide a more comprehensive scientific basis for early prevention, diagnosis, and treatment.

## 5. Strengths and limitations

This study utilized the MR method to effectively eliminate common confounding factors found in traditional observational studies, such as age, sex, lifestyle, and chronic inflammatory states, thereby providing a more accurate assessment of the causal relationship between BMD and stable angina. It offers new evidence for the causal link between BMD and stable angina. The study not only analyzed the impact of BMD on stable angina but also conducted reverse MR analysis to explore the influence of stable angina on BMD, explaining the potential reverse causality in the relationship between reduced BMD and the increased incidence of stable angina found in previous observational studies, and enriching our understanding of the complex relationship between osteoporosis and cardiovascular diseases. However, since the selected GWAS data primarily come from European samples, the generalizability of the study conclusions requires further clinical validation, especially in different racial and regional populations. The etiologies of elevated BMD are diverse. While this study established the association solely through MR, future prospective studies are required to clarify the underlying mechanisms. Additionally, once BMD GWAS databases are further enriched, stratified MR analyses could be conducted to identify specific thresholds at which BMD exerts its effects. Subsequent research should also employ larger sample sizes and more representative populations to enhance the generalizability of the findings.

## 6. Conclusions

In summary, this study innovatively employed the MR method to investigate the causal relationship between BMD at various sites and stable angina. The results indicate a significant positive causal relationship between BMD, especially left heel BMD, and stable angina. Furthermore, there is a significant reverse causal relationship between stable angina and both left and right heel BMD. Clinicians should consider the level of BMD, particularly left heel BMD, when assessing a patient’s risk of cardiovascular disease. For individuals with increased BMD, further assessment should be made to determine the presence of pathological factors, and targeted intervention measures should be implemented to reduce the risk of cardiovascular disease. For patients with stable angina, attention should be paid to changes in BMD, and appropriate measures such as increasing PA and supplementing with calcium should be taken to prevent the occurrence of osteoporosis.

## Author contributions

**Conceptualization:** Yuewei Song.

**Data curation:** Yuewei Song, Hao Tian Li, Yaxin Pan.

**Formal analysis:** Hao Tian Li, Yaxin Pan.

**Supervision:** Yanjun Liu, Weidong Sun.

**Validation:** Yanjun Liu, Weidong Sun.

**Writing – original draft:** Yuewei Song, Hao Tian Li.

**Writing – review & editing:** Yanjun Liu, Weidong Sun.

## Supplementary Material


