# Supplementary material for: Causal relationship between stable angina and bone mineral density: A two-sample bidirectional Mendelian randomization study
Source: Medicine (Baltimore). 2025 Nov 7;104(45):e45799. doi: 10.1097/MD.0000000000045799 (PMC12599691; doi:10.1097/MD.0000000000045799)
Supplement: Supplementary file 1 [file medi-104-e45799-s001.pdf]

Supplementary Figure 1.

(A) MR leave-one-out sensitivity analysis for BMD (right) and left heel BMD (left) on stable angina pectoris (SAP).

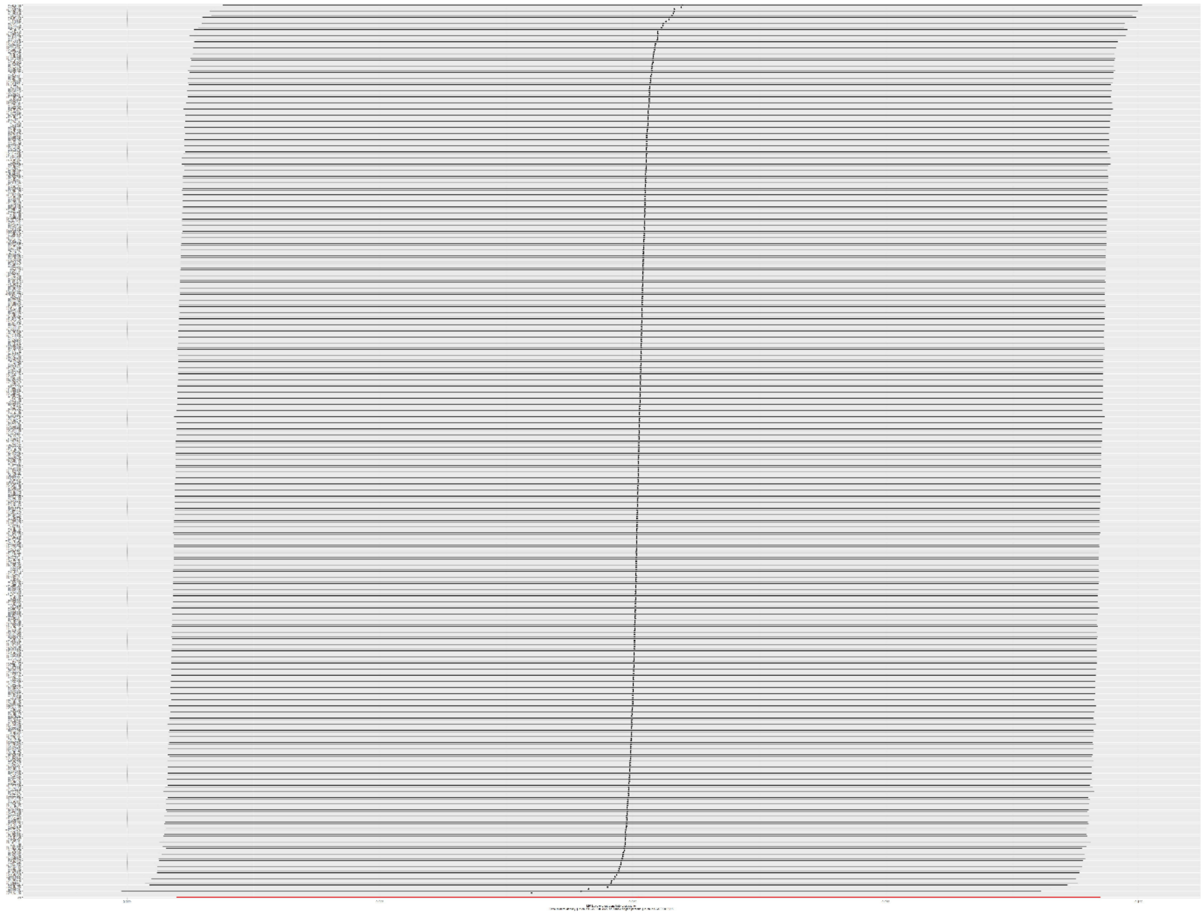

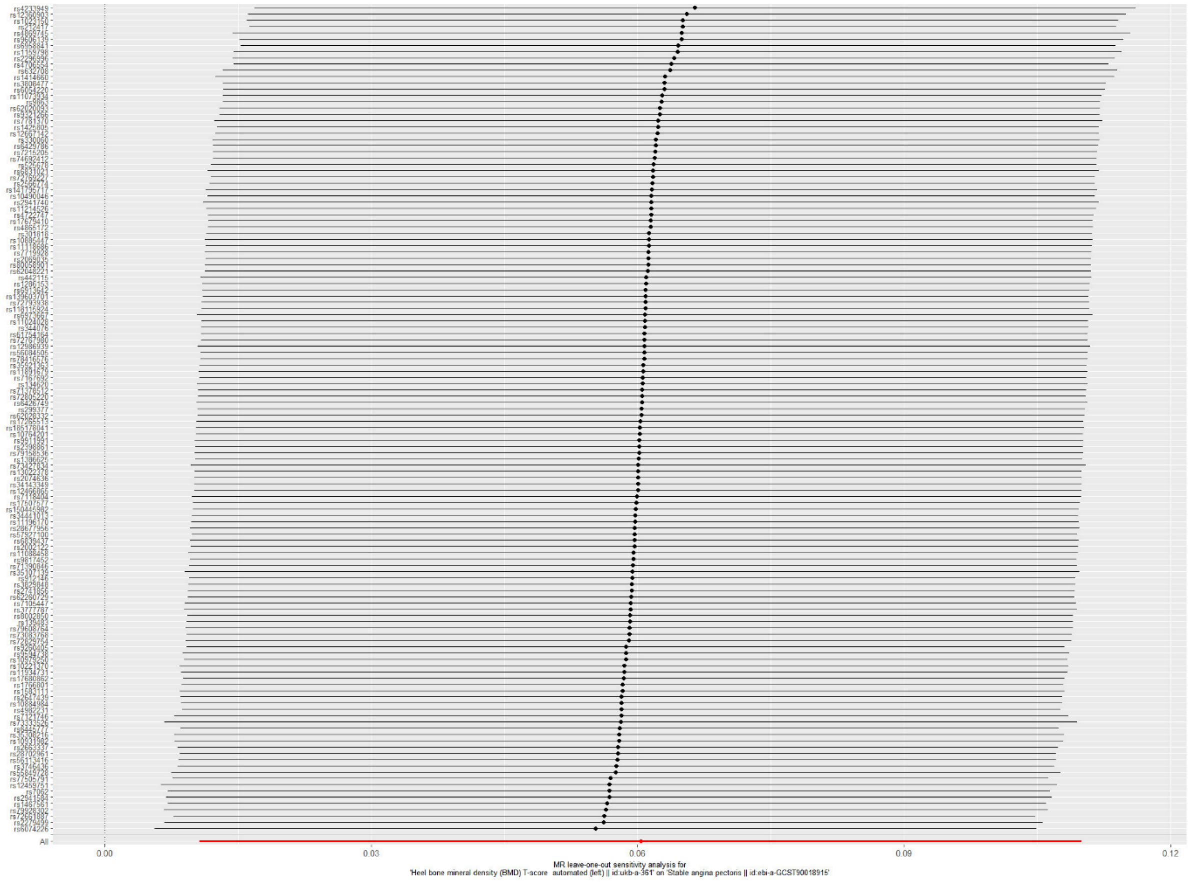

(B) Funnel plot of the IVW and MR-Egger models for BMD (right) and left heel BMD (left) with potential association with stable angina pectoris (SAP).

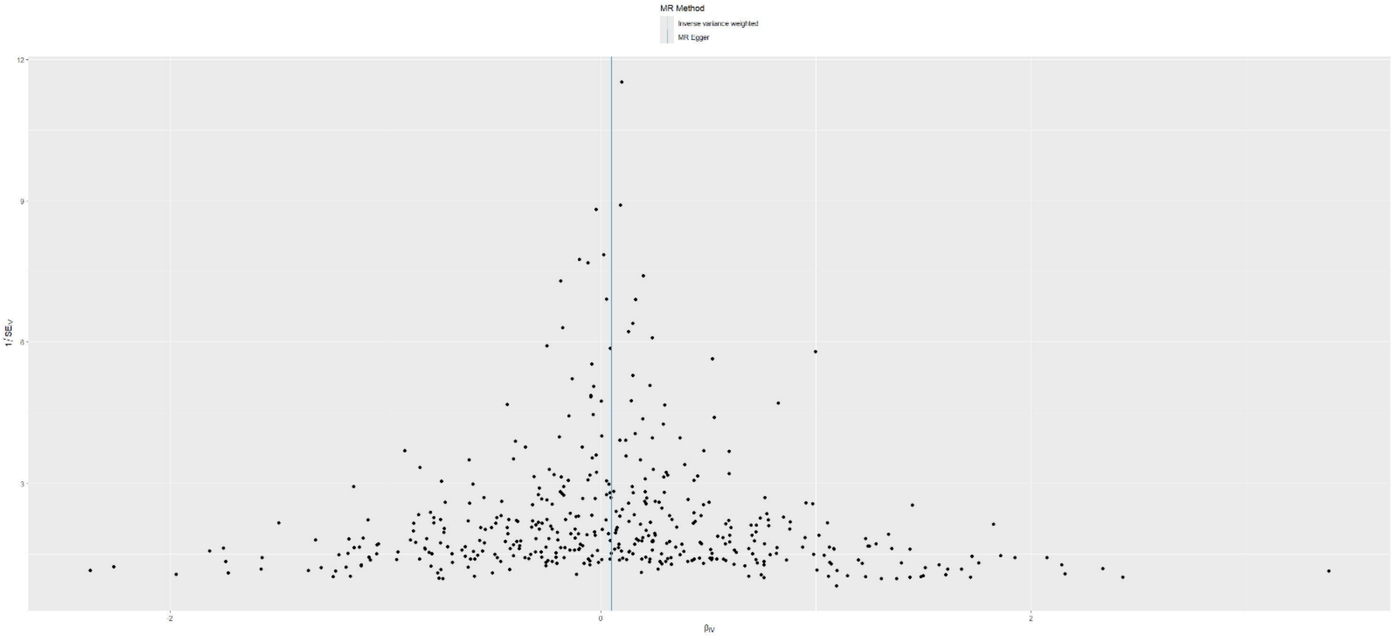

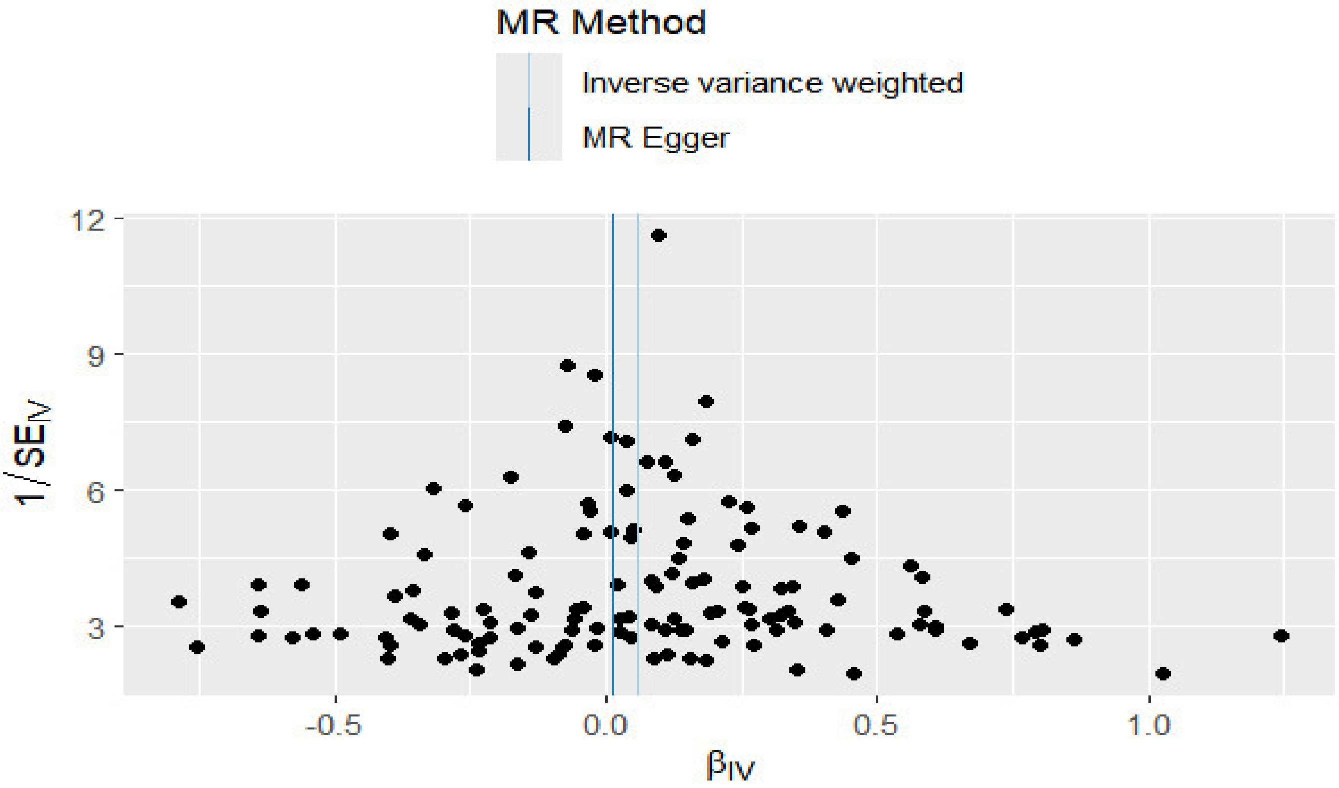

(C) Scatter plot of BMD (right） and left heel BMD (left) with potential association with stable angina pectoris (SAP).

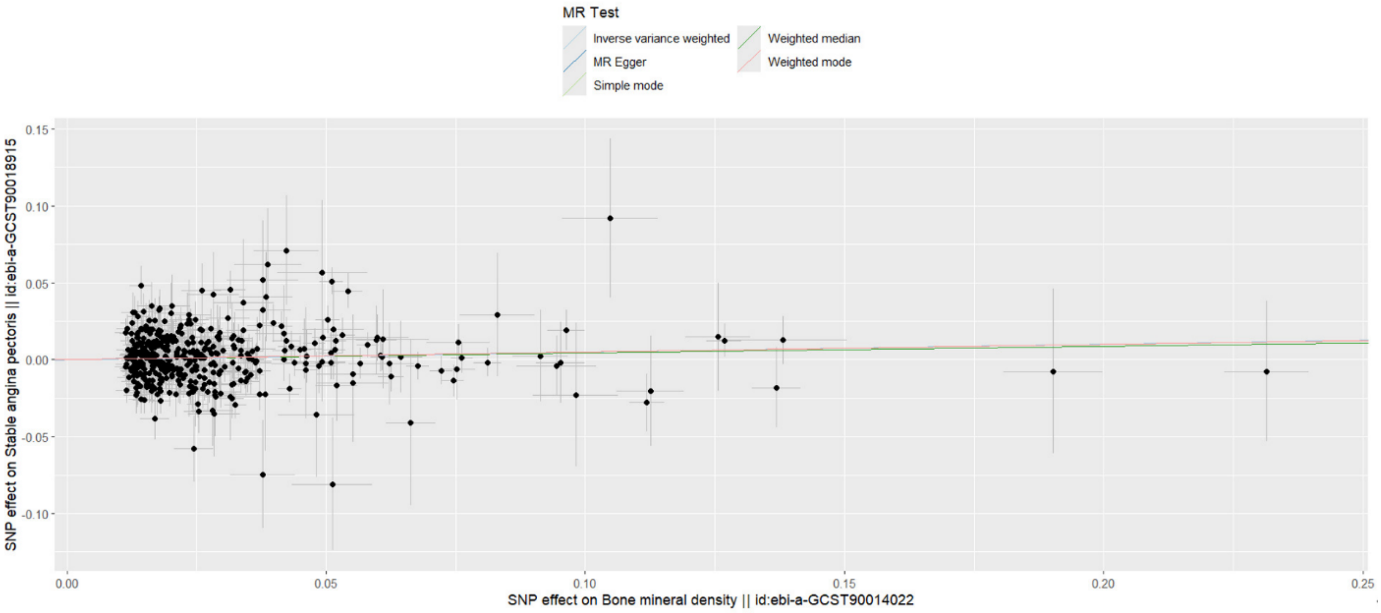

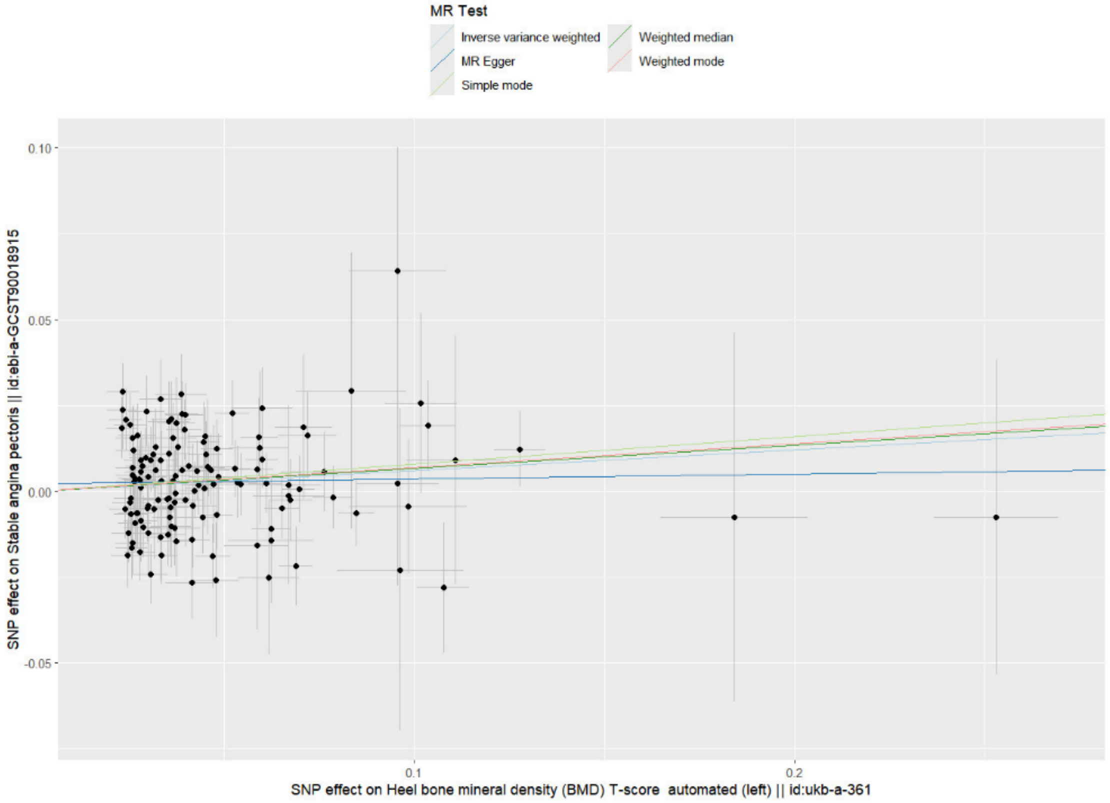

(D) Forest plot of the causal effects of SNPs associated with BMD (right) and left heel BMD (left) on stable angina pectoris (SAP).

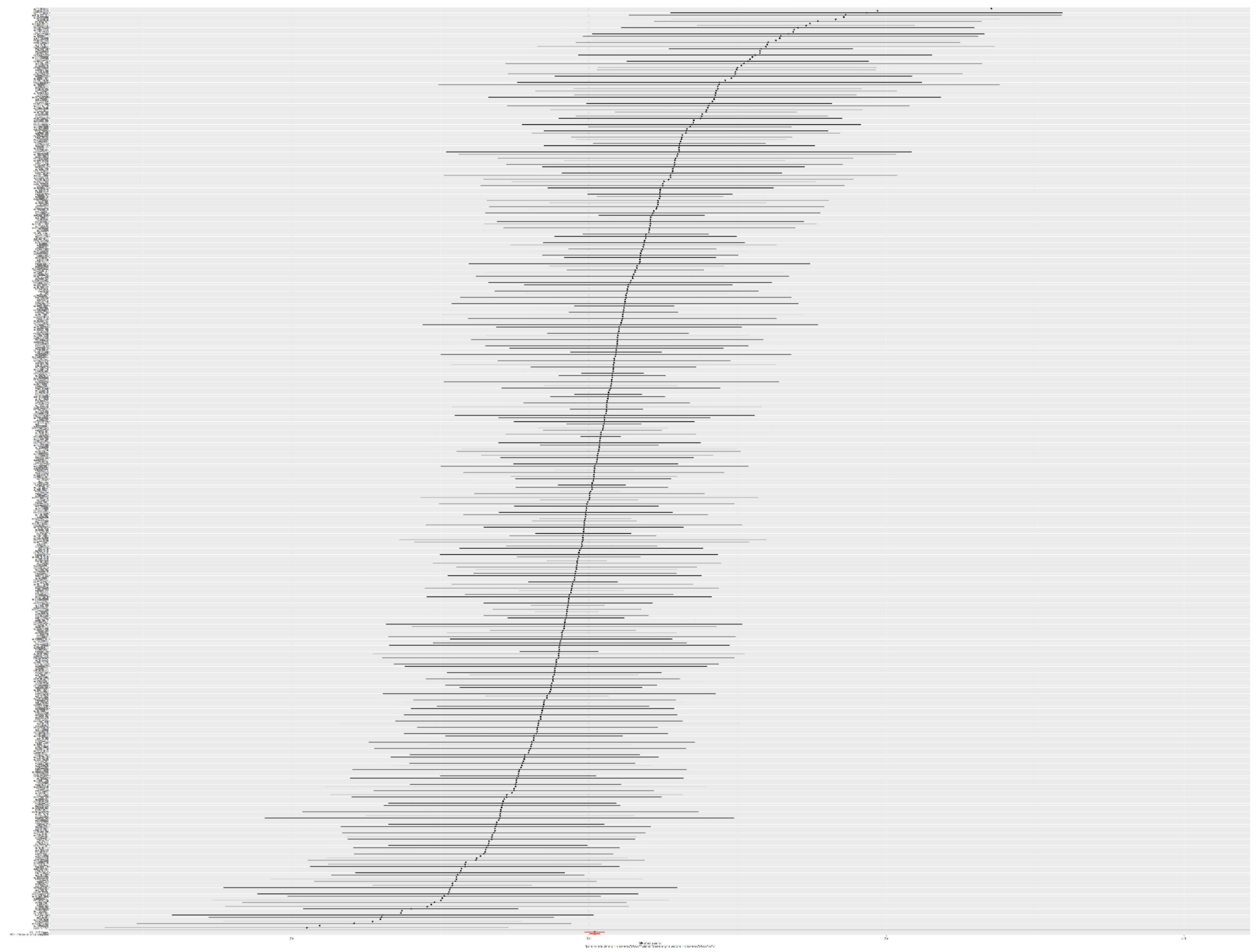

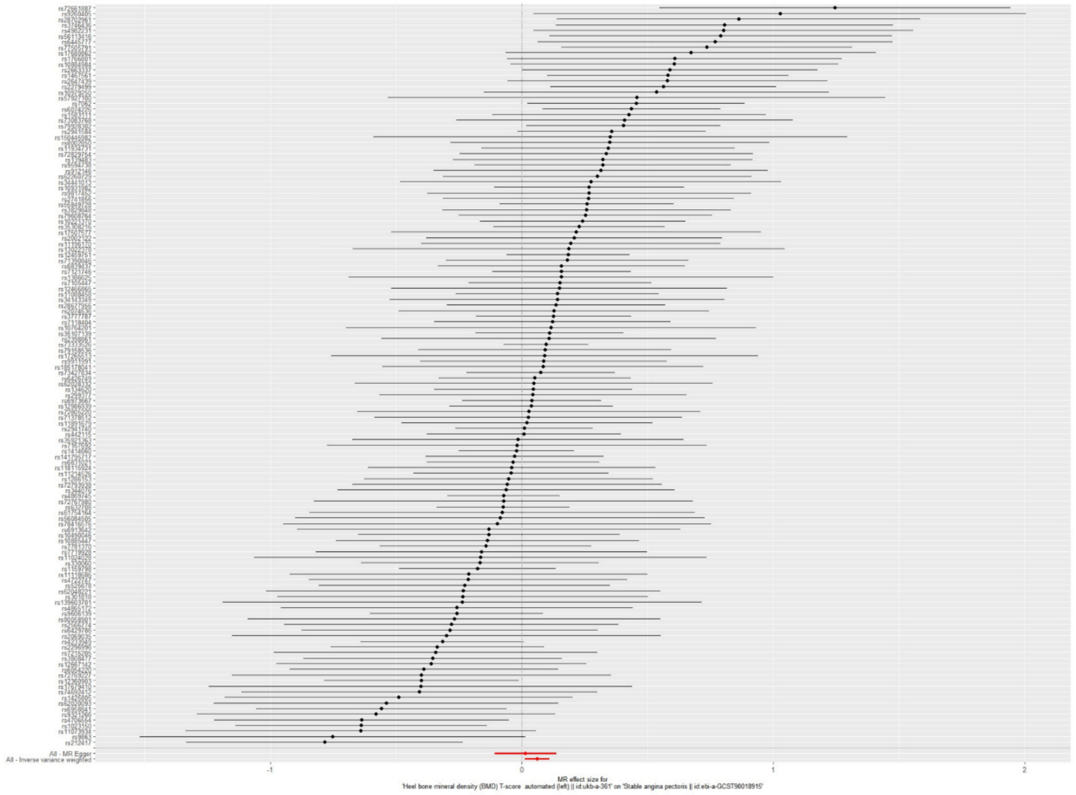

Supplementary Figure 2.

(A) Mendelian Randomization (MR) leave-one-out sensitivity analysis for stable angina pectoris (SAP) with left heel BMD (left) and right heel BMD (right).

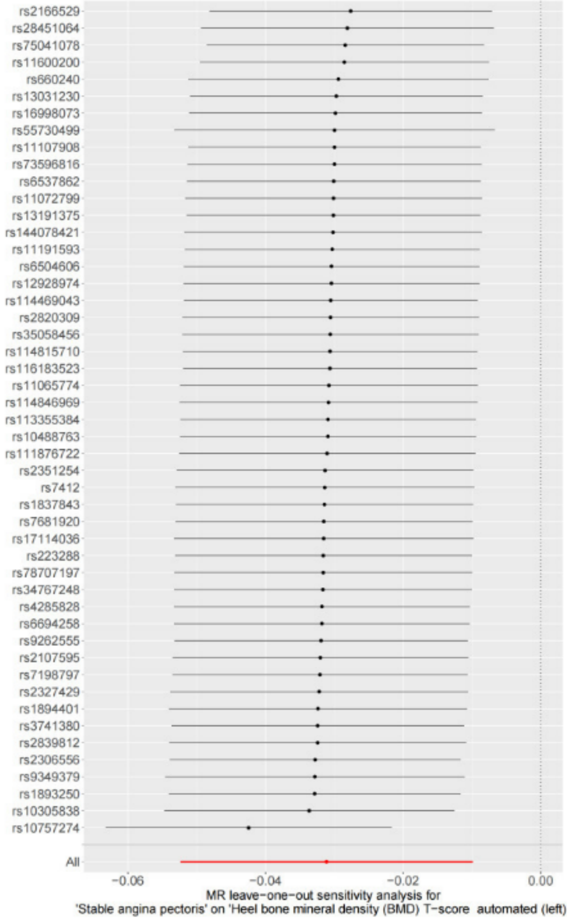

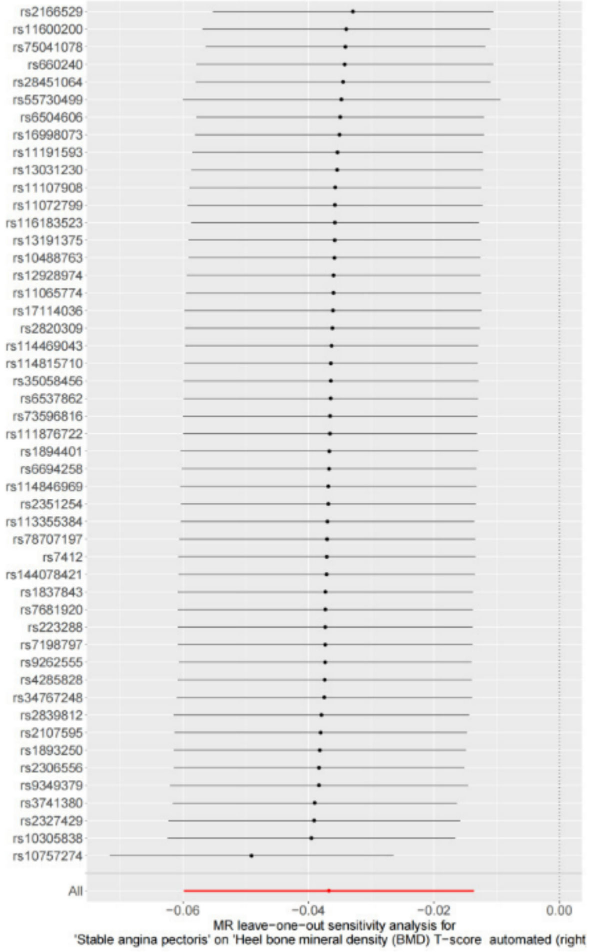

(B) Funnel plot of the Inverse Variance Weighted (IVW) and MR-Egger models for the association between stable angina pectoris (SAP) and left heel BMD (left) and right heel BMD (right).

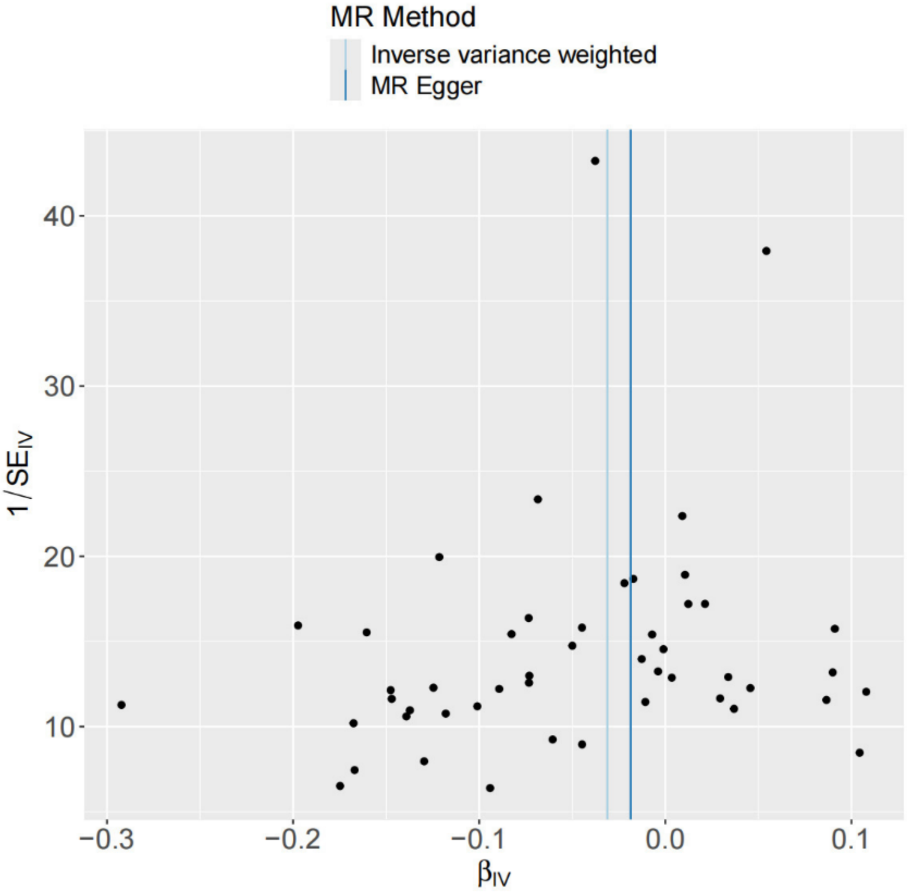

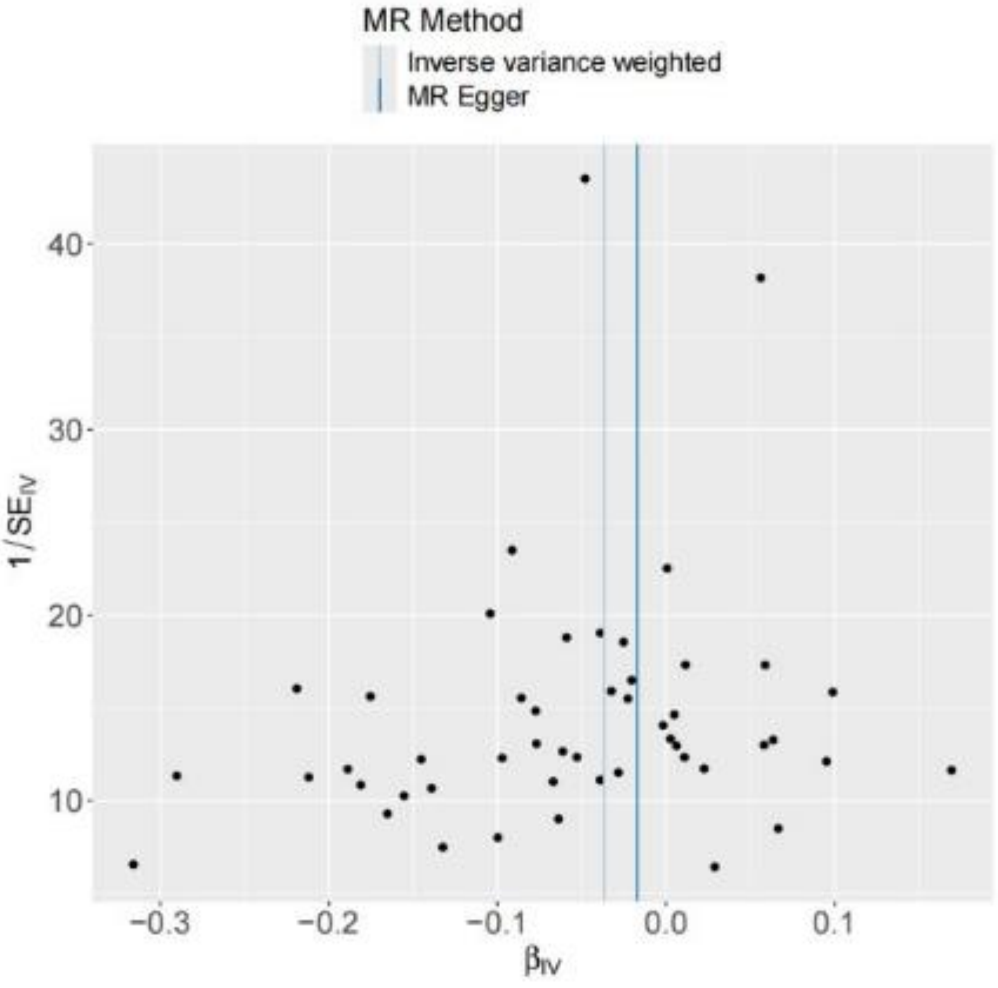

(C) Scatter plot of the relationship between stable angina pectoris (SAP) and left heel BMD (left) and right heel BMD (right).

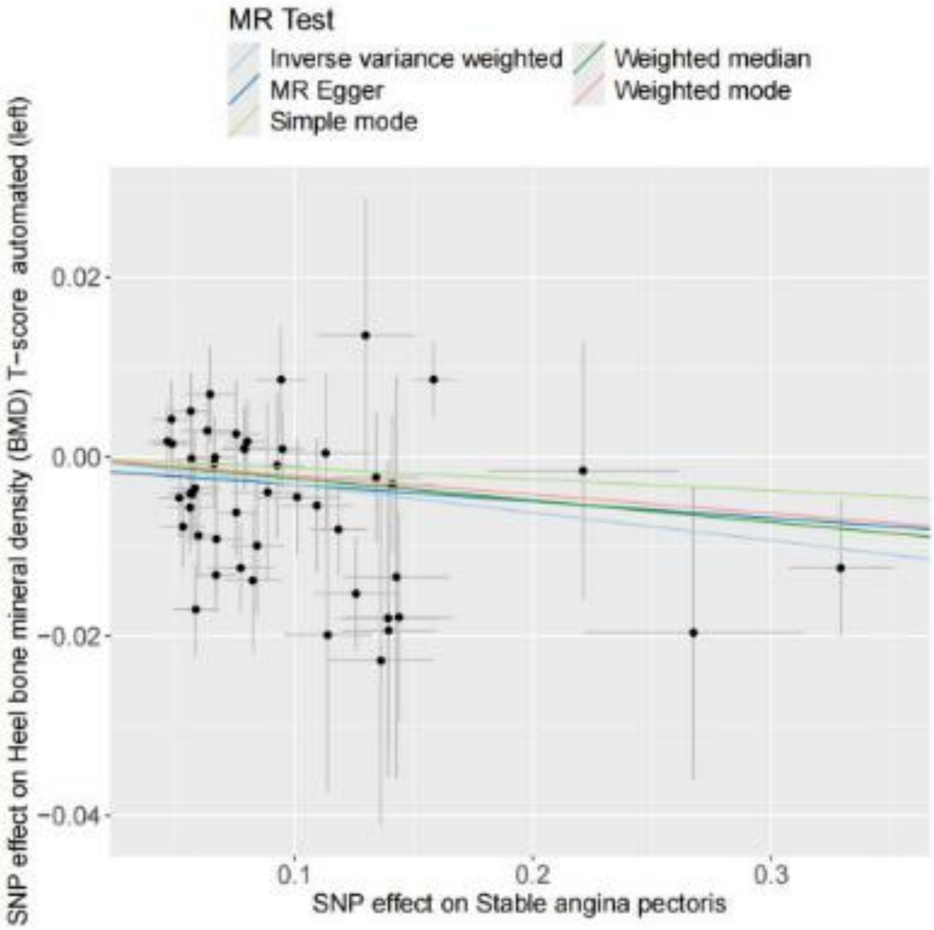

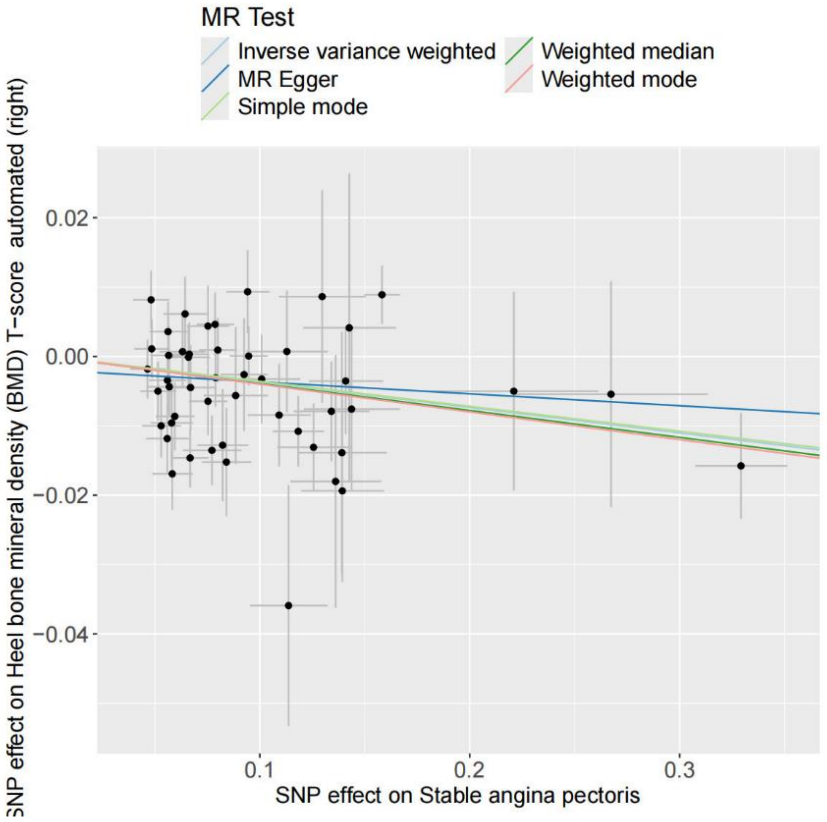

(D) Forest plot of the association between stable angina pectoris (SAP) and left heel BMD (left) and right heel BMD (right).

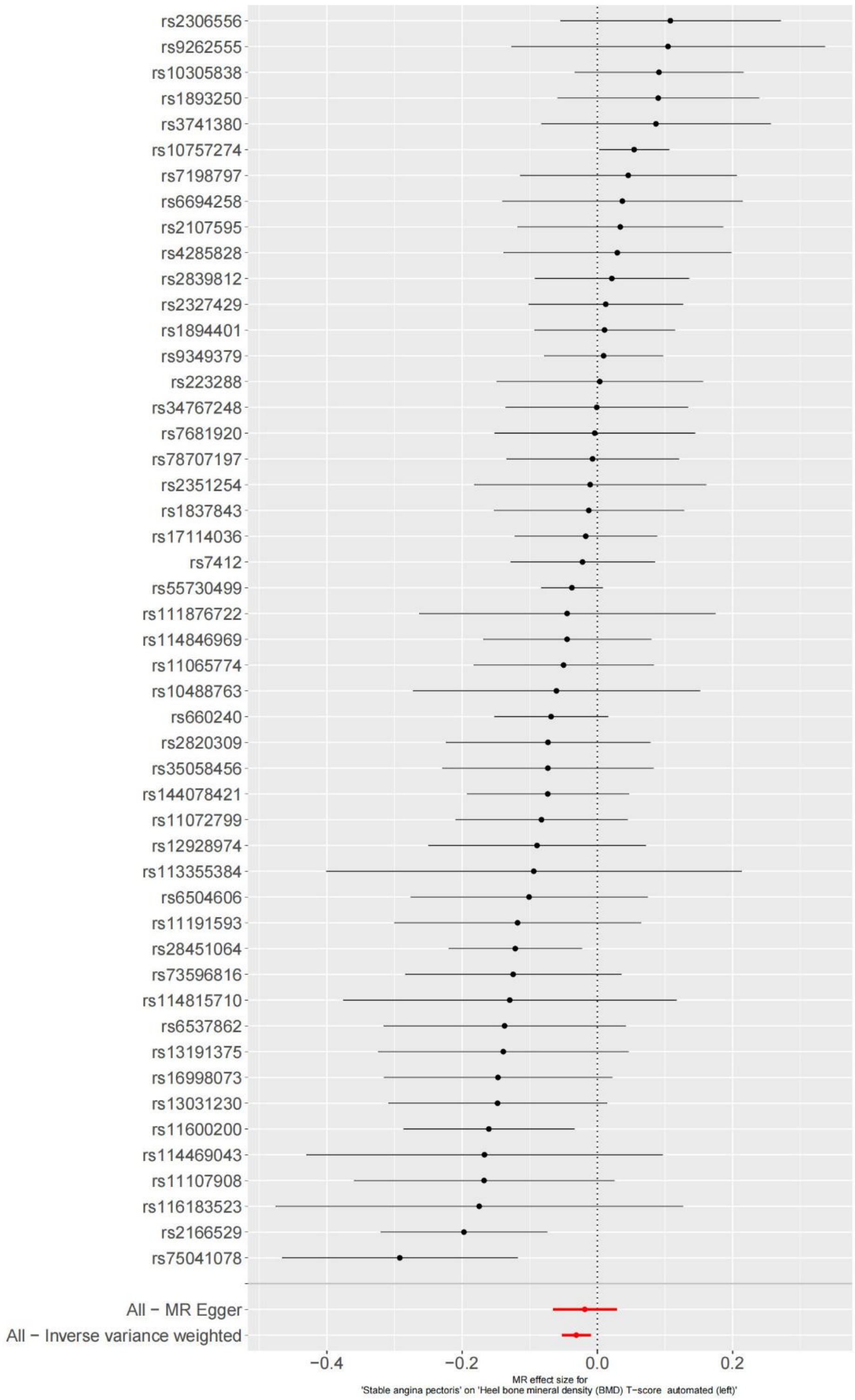

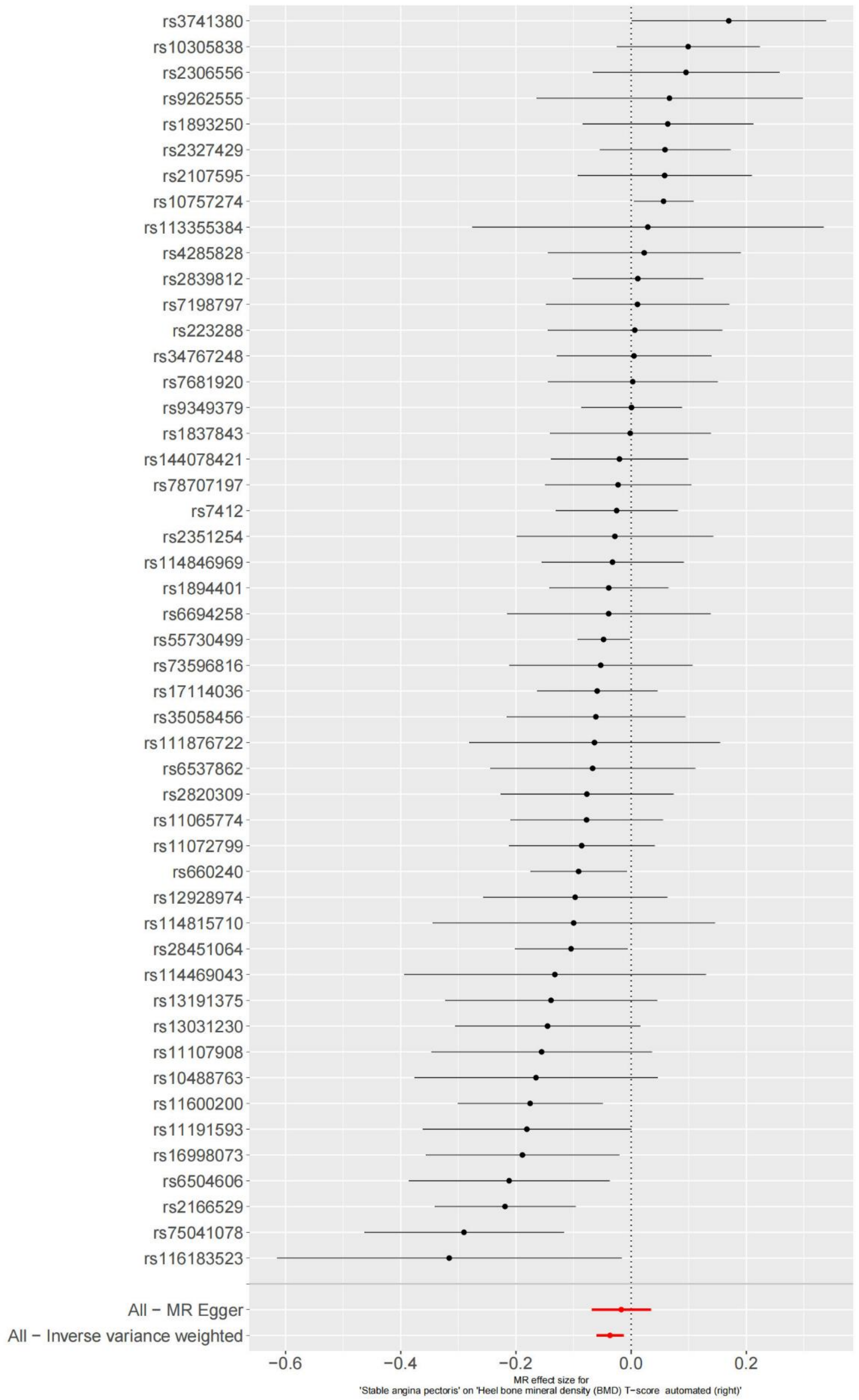
rs3741380
rs10305838
rs2306556
rs9262555
rs1893250
rs2327429
rs2107595
rs10757274
rs113355384
rs4285828
rs2839812
rs7198797
rs223288
rs34767248
rs7681920
rs9349379
rs1837843
rs144078421
rs78707197
rs7412
rs2351254
rs114846969
rs1894401
rs6694258
rs55730499
rs73596816
rs17114036
rs35058456
rs111876722
rs6537862
rs2820309
rs11065774
rs11072799
rs660240
rs12928974
rs114815710
rs28451064
rs114469043
rs13191375
rs13031230
rs11107908
rs10488763
rs11600200
rs11191593
rs16998073
rs6504606
rs2166529
rs75041078
rs116183523
All − MR Egger
All − Inverse variance weighted
−0.6
−0.4
−0.2
0.0
0.2
MR effect size for
'Stable angina pectoris' on 'Heel bone mineral density (BMD) T−score automated (right)'
